# Delayed Diagnosis of Pemphigus Vulgaris Initially Presenting as an Oral Ulcer: A Case Report

**DOI:** 10.31729/jnma.7594

**Published:** 2022-07-31

**Authors:** Ramesh Lamichhane, Saroj Chaudhary

**Affiliations:** 1 Department of Internal Medicine, Jalalabad Ragib Rabeya Medical College, Pathantula, Sylhet, Bangladesh; 2 Department of Cardiology, Nepal Cardio Diabetes and Thyroid Center, Budhanilkantha, Kathmandu, Nepal

**Keywords:** *delayed diagnosis*, *oral ulcer*, *pemphigus vulgaris*

## Abstract

Pemphigus vulgaris is a rare autoimmune mucocutaneous blistering disease clinically presenting as vesicles, bullae, and erosion and histologically characterized by suprabasal split and acantholysis. It usually affects mucous membranes and skin. Recurrent oral ulcers can only be the clinical manifestation before progressing into skin lesions. This can lead to the delayed diagnosis of this disease. Here we report a case of pemphigus vulgaris which was diagnosed after years of suffering from an oral ulcer that eventually progressed to widespread skin blistering and ulceration. The patient was treated with oral prednisolone which showed improvement within a week. Physicians should consider the differential diagnosis of pemphigus vulgaris in patients presenting with a recurrent oral ulcer.

## INTRODUCTION

Pemphigus includes a group of chronic autoimmune blistering diseases. Pemphigus vulgaris is the commonest one that affects the skin and mucous membrane. The oral cavity is the most commonly involved and initial site of this disease.^1^ Recurrent oral ulcer can be the only early manifestation of this disease which can lead to diagnostic confusion and delay in management.^2^ We present the case of full-blown cutaneous lesion of pemphigus vulgaris after one and half years of recurrent oral ulcer.

## CASE REPORT

**Figure 1:**
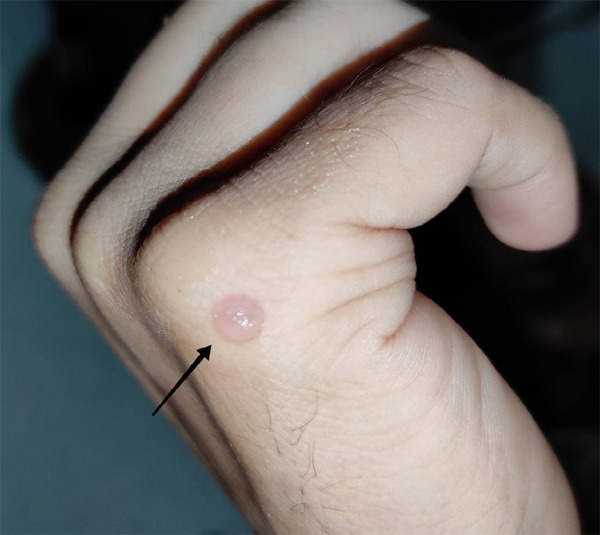
Clear fluid-filled blister.

A 27-year-old male patient presented with chief complaints of fluid-filled blisters on the skin (Figure 1) followed by ulceration involving widespread areas of the body for 3 months. On the careful assessment of past history, the patient was suffering from a painful oral ulcer for one and half years which caused him difficulty swallowing. He had suffered a weight loss of 8 kg due to limited intake of food due to painful swallowing. Other comorbidities include diabetes mellitus for 2 years controlled with insulin. Past dental and personal history was non-contributory. He did receive antiseptic gel and oral analgesics for oral ulcers without any improvement.

**Figure 2:**
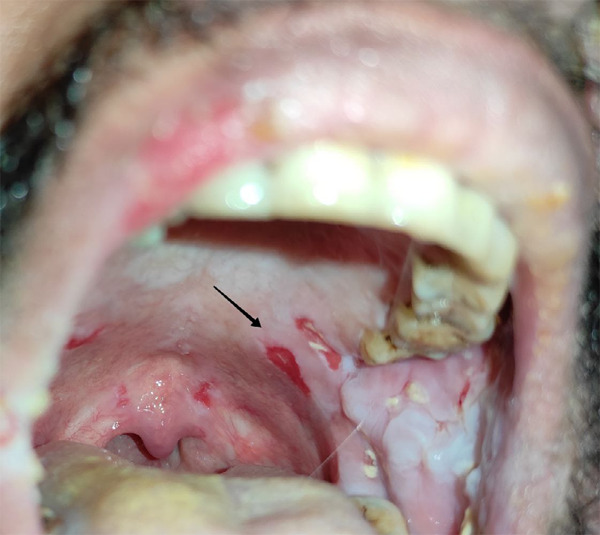
Oral cavity ulcers in soft and hard palate.

On general examination, the patient is moderately built, and all his vital signs were within normal limits. On oral examination, there were multiple intraoral ulcers over the soft palate, and hard palate (Figure 2).

**Figure 3:**
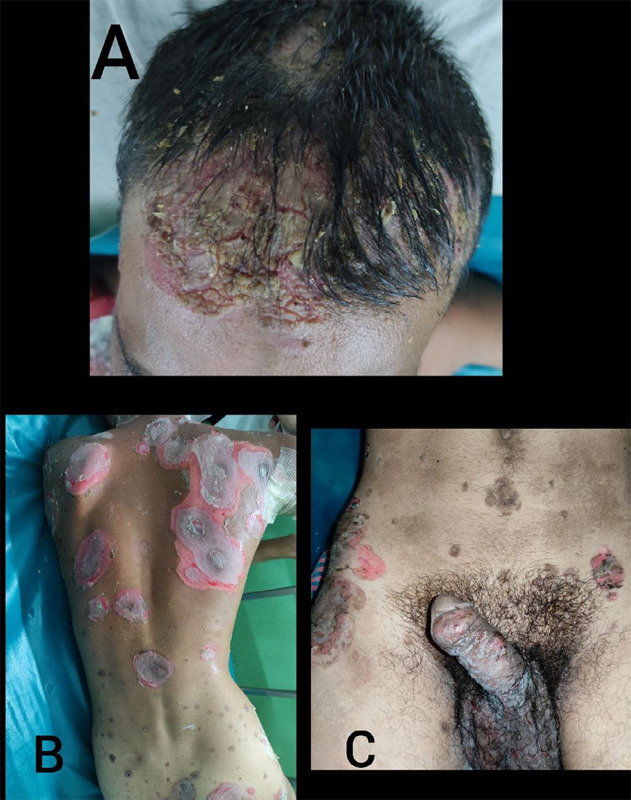
A) Ruptured blister with crust in scalp, B) Ruptured blister in Back and C) Ruptured blisters in Penis.

Fluid-filled vesicles were not seen in the mouth. There were widespread ulcers covered with crusts over the whole body including the scalp, back and genitalia (Figure 3 A, B, C). Nikolsky's sign was positive.

A provisional diagnosis of pemphigus vulgaris was made. The list of differential diagnoses included bullous pemphigoid and bullous systemic lupus erythematosus (SLE). A skin biopsy of the lesion was performed from the left lower leg under local anesthesia and sent for histopathology. Histopathological examination revealed suprabasal split and acantholysis with few inflammatory cells. Based on clinical and histopathological findings a final diagnosis of pemphigus vulgaris was made. Antinuclear antibody was negative.

The patient was treated with oral prednisolone 1mg/kg/ day. The patient showed improvement within weeks of starting treatment. The patient was discharged on the 10^th^ day on request and was scheduled for follow-up after 2 weeks. Gradual tapering of the steroid dose was planned. He didn't come for further follow-up care in our institution.

## DISCUSSION

Pemphigus vulgaris is a life-threatening mucocutaneous blistering disease. This disease is rare with a global incidence of 0.1-0.5 per 100,000 per year with a higher incidence in Ashkenazi Jews, Japanese, and populations of Mediterranean ancestry.^3^ The disease is mediated by circulating immunoglobulin G autoantibodies against the epithelial cell junction called desmosomal cadherin, desmoglein 1 and 3, resulting in loss of cell adhesion called acantholysis.^4^

Pemphigus vulgaris predominantly affects the skin and mucus membrane. The mucosa is almost always involved. Oral mucosa is the most common site. The majority of patients only have oral ulcers for which they visit medical professionals.^5^ This disease can be diagnosed at an early stage but still, diagnostic delays are common in oral pemphigus vulgaris.^6^ Patients can only have oral ulcers which may cause a delay in diagnosis of this potentially life-threatening condition. The oral ulcer is then followed by skin lesions in most cases.^2^ Skin involvement causes flaccid blister with clear fluid which eventually ruptures resulting in painful erosion which will be covered by a crust that lacks the tendency to heal. Our patient also had an oral ulcer for one and a half years which then progressed to a skin lesion. Our patient had very few intact blisters and the majority of blisters were ruptured and covered with crusts.

Pemphigus vulgaris typically affects 40-60 years of age.^7^ Our patient was only 27 years when he was affected. This suggests that we should consider the diagnosis even at an early age. Nikolsky's sign is positive in pemphigus vulgaris which means applying lateral pressure on an intact blister will result in separation of the normal epidermis and extension of the blister.^8^ Nikolsky's sign was positive in our present case. Histopathologic findings in the pemphigus vulgaris are suprabasal split and acantholysis which was seen in our case.

Pemphigus vulgaris has a mortality rate of 60-90% without treatment.^9^ Life-threatening complications like sepsis, fluid, and electrolyte imbalance may occur. Corticosteroid is the mainstay treatment of this disease which was used in this case. Alternative treatment includes rituximab and adjuvant therapy like mycophenolate mofetil or azathioprine. One of the trial suggested that combination of rituximab and prednisolone over the prednisolone alone as first-line therapy for pemphigus vulgaris.^10^ The high cost of rituximab limited the use of rituximab in our case. Our patient responded to prednisolone monotherapy.

The delay in the progression from oral to skin lesion made the diagnosis late in our case. An early diagnosis while the patient was suffering from an oral ulcer may have prevented the progression of disease to the cutaneous lesion. All physicians especially dental and otolaryngology doctors should keep differential diagnosis of pemphigus vulgaris in patient presenting with recurrent oral ulcer even at an early age.
